# Thermally controlled widening of droplet etched nanoholes

**DOI:** 10.1186/1556-276X-9-285

**Published:** 2014-06-09

**Authors:** Christian Heyn, Sandra Schnüll, David E Jesson, Wolfgang Hansen

**Affiliations:** 1 Institut für Angewandte Physik und Zentrum für Mikrostrukturforschung, Jungiusstraße 11, Hamburg 20355, Germany; 2 School of Physics and Astronomy, Cardiff University, Cardiff CF24 3AA, UK

**Keywords:** Semiconductor, Nanostructuring, Self-assembly, Droplet etching

## Abstract

We describe a method to control the shape of nanoholes in GaAs (001) which combines the technique of local droplet etching using Ga droplets with long-time thermal annealing. The cone-like shape of inverted nanoholes formed by droplet etching is transformed during long-time annealing into widened holes with flat bottoms and reduced depth. This is qualitatively understood using a simplified model of mass transport incorporating surface diffusion and evaporation. The hole diameter can be thermally controlled by varying the annealing time or annealing temperature which provides a method for tuning template morphology for subsequent nanostructure nucleation. We also demonstrate the integration of the combined droplet/thermal etching process with heteroepitaxy by the thermal control of hole depth in AlGaAs layers.

## Background

**Figure 1:**
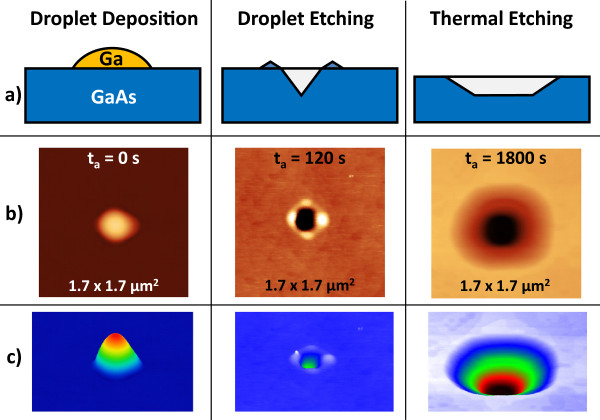
**Schematic of the droplet etching process and AFM images. (a)** Schematic of the combined droplet and thermal etching process with deposition of Ga as droplet material during 2.5-s deposition time, droplet etching up to removal of the droplet material, and subsequent thermal etching during long-time annealing. **(b)** 1.7 ×1.7 µm^2^ top-view AFM micrographs illustrating the different stages for *T* = 650℃. The as-grown droplets with average height of 120 nm are visible at zero annealing time *t*_a_= 0 s. At *t*_a_= 120 s, all droplet material has been removed and nanoholes with average depth of 68 nm have been formed. After *t*_a_ = 1,800 s, the hole width has been substantially increased by thermal etching. **(c)** Color-coded perspective AFM images of the micrographs from **(b)**.

**Figure 2:**
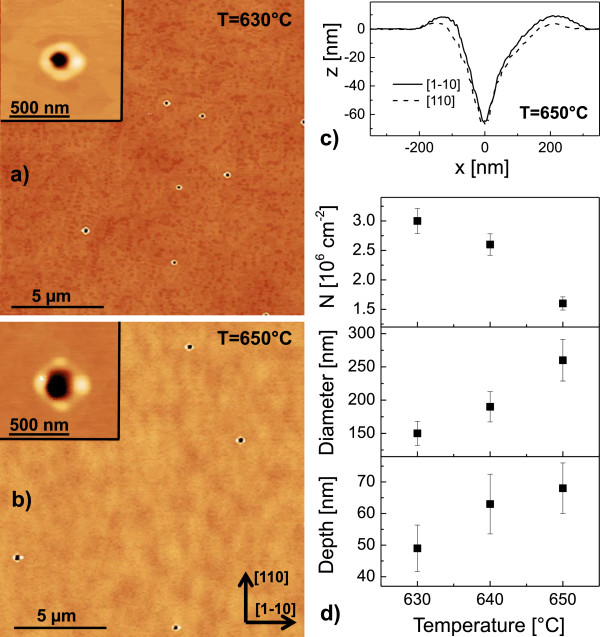
**GaAs surfaces after Ga-LDE at temperatures above the GaAs congruent evaporation temperature.** The Ga droplet material coverage is 2.0 ML and the annealing time *t*_a_= 120 s. **(a)** AFM images of LDE nanoholes for etching at *T* = 630℃. **(b)** AFM images of LDE nanoholes for etching at *T* = 650℃. **(c)** Linescans of a nanohole from **(b)**. **(d)** Average hole density *N*, diameter and depth as function of the process temperature. The hole diameter is taken at the plane of the flat surface, and the hole depth is defined as the distance between the flat surface plane and the deepest point of the hole.

The self-assembled patterning of semiconductor surfaces by liquid metal droplets [1-6] has been established as an important technique for the fabrication of novel semiconductor nanostructures. This so-called local droplet etching (LDE) is fully compatible with the demanding requirements of molecular beam epitaxy (MBE) and can be integrated into the growth of semiconductor heterostructures. The utilization of metal droplets during semiconductor epitaxy has a long tradition, starting in 1990, when Chikyow and Koguchi established droplet epitaxy [7]. There, the metal droplets are crystallized under, e.g, an As flux for the fabrication of semiconductor quantum dots or rings [8-12]. In contrast to droplet epitaxy, droplet etching takes place at significantly higher temperatures and low As flux. This process drills nanoholes into the substrate which are surrounded by walls crystallized from arsenides of the droplet material [13]. A schematic of the droplet etching process is shown in Figure 1a, and typical atomic force microscopy (AFM) images of surfaces with droplet etched nanoholes are contained in Figures 2a,b.

Nanoholes drilled by LDE can be filled with a material different from that of the substrate and so have several important advantages for the self-assembly of quantum structures. For example, this allows the creation of strain-free GaAs quantum dots [14-16] with the capability to precisely adjust the dot size by filling the holes only partially. Furthermore, the realization of ultra-short nanopillars [17] has been demonstrated. In particular, the nanopillars represent a novel type of nanostructure for studies of one-dimensional thermal [18] or electrical [19] transport.

The process of droplet etching is performed in two steps. First, Ga is deposited and self-assembled Ga droplets are formed in the Volmer-Weber growth mode [20]. In a second post-growth thermal annealing step, the initial droplets are transformed into nanoholes. Diffusion of As from the GaAs substrate into Ga droplets, driven by a concentration gradient, is the central process for droplet etching [13]. This is accompanied by removal of the droplet material, probably by detachment of Ga atoms from the droplets and spreading over the substrate surface [19]. In this work, the influence of long-time annealing on the morphology of the nanoholes is studied at temperatures above the GaAs congruent evaporation temperature [21] where we demonstrate that thermal desorption and surface mass transport of substrate material becomes relevant.

## Methods

The samples discussed here are fabricated using solid-source molecular beam epitaxy on (001) GaAs substrates with a valved cracker cell for As_4_ supply. The Ga flux is adjusted for a GaAs growth rate of 0.8 monolayers (ML)/s. The As flux during GaAs buffer layer growth corresponds to a flux gauge reading of 1 ×10^−5^ Torr. During droplet etching, the As flux is minimized to less than 1 ×10^−7^ Torr by closing the As valve, the As cell shutter and in addition the main shutter in front of the sample during annealing.

After growth of a 100-nm-thick GaAs buffer layer at a temperature *T* = 600℃ to smooth the surface, the As shutter and valve are closed and the temperature is increased to the annealing temperature of 630℃ to 670℃. Ga is the deposited for 2.5 s corresponding to a droplet material coverage *θ*= 2.0 ML. After deposition of the droplet material, the initial droplets are transformed into nanoholes during post-growth annealing for a time *t*_a_. After annealing, the samples are quenched by switching off the substrate heater. Figure 1a shows a sketch of the whole process including the shape modification of the droplet etched nanoholes during long-time annealing, and Figure 1b,c displays typical atomic force microscopy (AFM) images visualizing the different stages.

## Results and discussions

The purpose of this study is to examine droplet etching processes at high temperature. Previously, the generation of nanoholes by LDE with Ga droplets has been demonstrated in the temperature regime between 570℃ and 620℃ [13]. Figure 2a,b establishes that droplet etching with Ga on GaAs is possible also above the congruent evaporation temperature of 625℃ [21,22]. The holes have an average depth of 68 nm at *T* = 650℃ (Figure 2c) which is more than four times deeper compared with previous Ga-LDE results [13].

A summary of the temperature-dependent structural characteristics of the nanoholes is plotted in Figure 2d. The hole density *N* decreases with *T* in accordance with previous results on Ga- [13] or Al-LDE [23]. A particularly interesting observation is that the holes have very low densities (≃10^6^ cm ^−2^). This demonstrates that high *T* droplet etching can be used to generate low-density nanohole templates for the subsequent creation of well-separated nano-objects following deposition. The hole diameter increases with *T*, which is related to the increasing volume of the initial droplets *V*≃*θ*/*N* at conditions with reduced density *N*. Also, the hole depth increases with *T*. This temperature-dependent trend of hole depth is in agreement with previous experimental results [13,23] and has been modelled by a simple scaling law with a temperature-dependent etching rate [23]. More elaborate models considering the nanohole morphology are discussed in [13,24].

**Figure 3:**
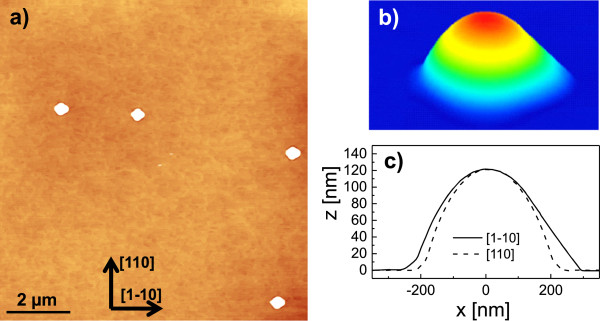
**GaAs surface with as-grown droplets. (a)** AFM micrograph of a GaAs surface with as-grown droplets after deposition of 2 ML Ga at *T* = 650℃ without annealing. **(b)** Color-coded perspective view of a single Ga droplet. **(c)** Linescans of the droplet from **(b)**. The average contact angle is 54°.

We now consider the influence of the annealing time *t*_a_ on nanohole morphology at constant temperature *T* = 650℃. Figure 3a,b shows Ga droplets on a GaAs surface prepared with immediate quenching of the sample after droplet deposition (*t*_a_= 0). The occurrence of Ga droplets at temperatures above the GaAs congruent evaporation temperature has already been studied previously [25,26], but there the droplets were formed by Langmuir evaporation. In the present samples, the droplet density of 1.9 ×10^6^ cm ^−2^ is almost equal to the nanohole density obtained at the same temperature (Figure 2d), which establishes that every initial droplet forms a nanohole. These droplets have an average height of 120 nm and average diameter of 470 nm (Figure 3c). This yields an average ratio between the droplet height and its radius of 0.51 ± 0.03 corresponding to a contact angle of 54°. Previous experiments [23] for Al-LDE on AlGaAs yielded a contact angle of 66°, which neither depends on temperature nor on droplet material coverage.

At *t*_a_= 120 s, all initial Ga droplets have been transformed into nanoholes with walls (Figure 2). This process is called local droplet etching and has already been studied previously [1,6,13]. The time during which droplet etching takes place is given by the time up to complete removal of the droplet material. Using a model of the LDE process described in [13], for Ga-LDE at *T* = 650℃, an etching time of 12 s is predicted. After this time, the droplet material is removed and droplet etching stops.

**Figure 4:**
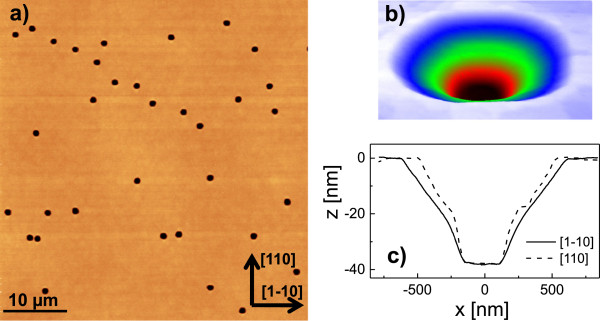
**GaAs surface with thermally widened holes. (a)** AFM micrograph of a GaAs surface with large thermally widened holes after Ga droplet etching and 1,800-s annealing at *T* = 650℃. **(b)** Color-coded perspective view of a single large hole. **(c)** Linescans of the hole from **(b)**.

A central result of this work is obtained during long-time annealing at high temperature where the droplet etched holes are observed to widen. Figure 4 shows an example of a sample prepared at *t*_a_= 1,800 s. Large holes are visible with an average diameter of the hole opening of 1,050 nm. The density of these large holes is 1.4 ×10^6^ cm ^−2^, which is almost equal to the density of droplet etched nanoholes obtained for *t*_a_= 120 s at the same temperature (Figure 2d). This supports our assumption that the large holes are modifications of the nanoholes drilled by droplet etching. Beyond the widening of the hole diameter, the long-time annealing also substantially modifies the shape of the holes. In detail, the side facet angle of the holes after droplet etching is in the range of 27° to 33°, whereas the average side facet angle of the large holes is about 5°. Furthermore, the bottom part of the inverted cone-like shaped LDE holes is rather peaked, whereas the large widened holes have a flat bottom plane of about 250 nm in diameter (Figure 4c). Finally, no walls are visible around the deep hole openings.

**Figure 5:**
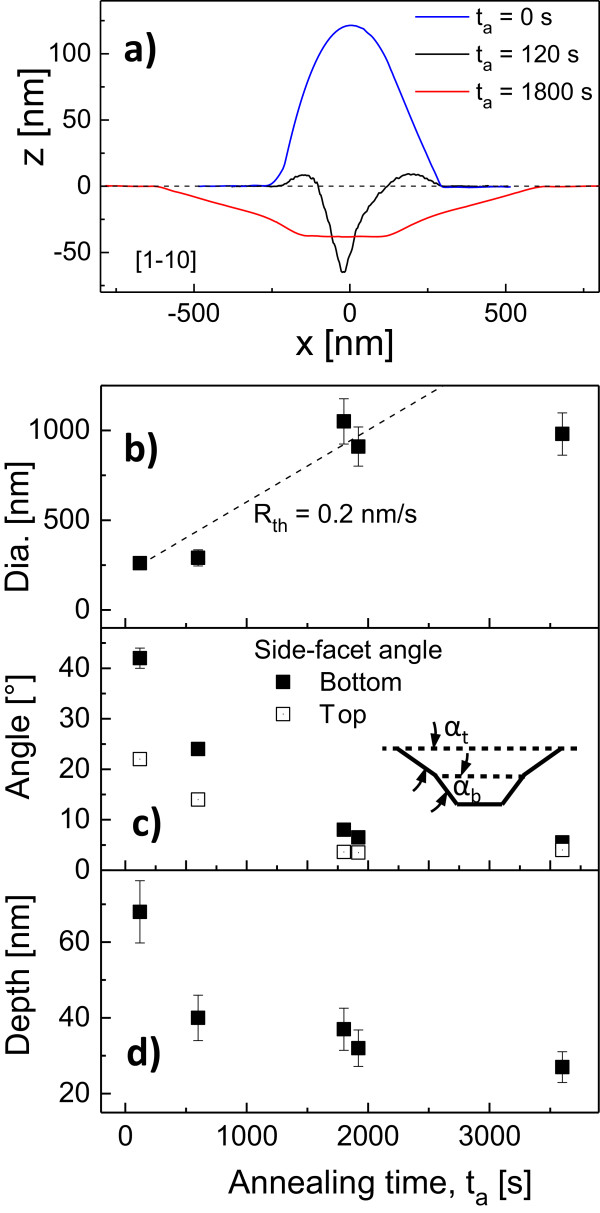
**Comparison of linescans and dependence of hole opening diameter, side facet angles and hole depth on *t***_**a**_**. (a)** Comparison of AFM linescans from an as-grown droplet (*t*_a_= 0 s, blue line), a nanohole after droplet etching (*t*_a_= 120 s, black line) and a thermally widened large hole (*t*_a_= 1,800 s, red line). Dependence of **(b)** the diameter of the hole opening, **(c)** the side facet angles at the bottom *α*_b_ and top *α*_t_ part of the holes and **(d)** of the hole depth on the annealing time *t*_a_. The dashed line in **(b)** corresponds to an estimated lateral etching rate of *R*_th_= 0.2 nm/s.

Figure 5a shows a direct comparison of typical AFM linescans from an as-grown droplet, a nanohole after droplet etching and a thermally widened large hole. The data confirm that the outer diameter of the walls around the droplet etched nanholes is almost equal to that of the initial droplets. This relationship has already been observed previously but at lower process temperatures [6].

The dependence of the hole opening diameter on the annealing time is plotted in Figure 5b. We observe an increasing diameter up to *t*_a_= 1,800 s followed by a saturation. The increasing hole opening diameter corresponds to a lateral etching rate of *R*_th_= 0.2 nm/s (Figure 5b). A saturation is also observed for the hole depth, which decreases up to *t*_a_= 1,800 s and saturates for higher *t*_a_ (Figure 5b).

**Figure 6:**
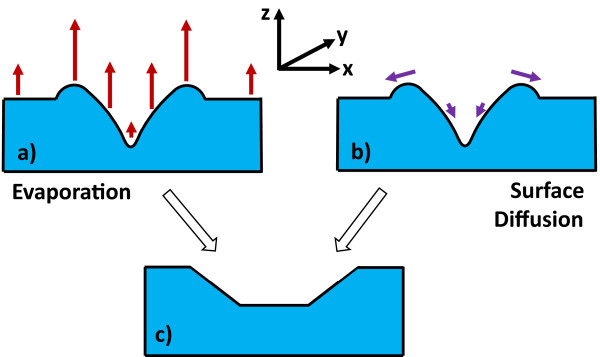
**Cross-sectional schematic of the proposed mass transport leading to thermally widened nanoholes shown in (c).** In **(a)**, the length of the arrows qualitatively represent the magnitude of material evaporation rates from various positions on the surface of a droplet etched nanohole. Similarly, in **(b)**, the length of the arrows qualitatively represent the magnitude of diffusive flux across the surface.

The evolution of nanoholes during annealing depends on surface mass transport processes which include direct evaporation and surface diffusion. Although such processes will depend in detail on the binary nature of GaAs, the main features of hole evolution can be qualitatively understood using standard models of surface evolution [27]. For simplicity, assuming isotropic surface energy, the chemical potential of the surface can be written as

(1) $$\mu =\mu_{0}+\Omega \gamma \left(\kappa_{x}+\kappa_{y}\right)$$

where *γ* is the isotropic surface energy, *Ω* is an atomic volume, and *κ*_*x*_ and *κ*_*y*_ are the two principal curvatures at a given position of the surface in *x* and *y* planes, respectively. Each curvature is taken to be positive for convex and negative for concave surfaces. *μ*_0_ is the reference chemical potential of the planar surface. In the case of direct evaporation into the vacuum, for small surface slopes, the removal of material from the surface will be proportional to the surface chemical potential in Equation 1. Figure 6a,b displays a schematic cross section of a nanohole formed by droplet etching, and Figure 6a schematically represents the magnitude of the expected evaporation rates based on the variation of *κ*_*x*_. This clearly predicts a reduction in the hole side-wall angle and a decrease in hole depth because of the reduced evaporation rate of the hole bottom compared with the planar surface, leading to the morphology in Figure 6c. For the case of mass transport by surface diffusion, the flux along the surface is given by

(2) J=−M∇μ

where *M* is the surface mobility. Figure 6b schematically represents the flux driven by gradients in chemical potential, and it can be seen that this also favours a decreasing hole side-wall angle and hole depth in agreement with the morphology in Figure 6c. Although anisotropic surface energy must also play an important role in the evolving morphology, this simple model of surface mass transport is qualitatively consistent with the general form of thermally widened holes, observed experimentally.

We therefore propose that long-time annealing a hole of a given size prepared by LDE will produce a final morphology which is approximately independent of annealing temperature (within the range studied) as the diameter, depth and side facet angles associated with the hole saturate with time (Figure 5). Although this might be consistent with our simple model of surface evolution for shallow surface profiles, evidence of faceting in Figure 5a suggests that surface energy anisotropy may also play a role in suppressing the hole morphology time evolution.

To study the influence of the process temperature on the widened holes, we have fabricated two additional samples both with *t*_a_= 1,800 s. For the first sample, a temperature of 650℃ was applied during droplet deposition and 670℃ during annealing. This sample has large holes with average diameter of 900 nm and average depth of 28 nm, which is in agreement with the samples fabricated at 650℃ and *t*_a_≥ 1,800 s shown in Figure 5b,c. This demonstrates that an elevated temperature during annealing alone does not modify the hole size. On the other hand, a sample fabricated at a temperature of 670℃ during both droplet deposition and annealing shows significantly larger holes with average opening diameter of 1,270 nm, average depth of 40 nm and flat bottom plane with 300-nm diameter. This finding indicates that the size of the droplet etched holes influences the size of the large holes after thermal treatment. For deposition and annealing at *T* = 650℃, droplet etched holes have a depth of 68 nm (Figure 2d). After 1,800-s long-time annealing, the depth is reduced to 35 nm, which is approximately half. For *T* = 670℃, droplet etched holes of about 80-nm depth are expected (Figure 2d). Here, the long-time annealing also approximately halves the depth.

**Figure 7:**
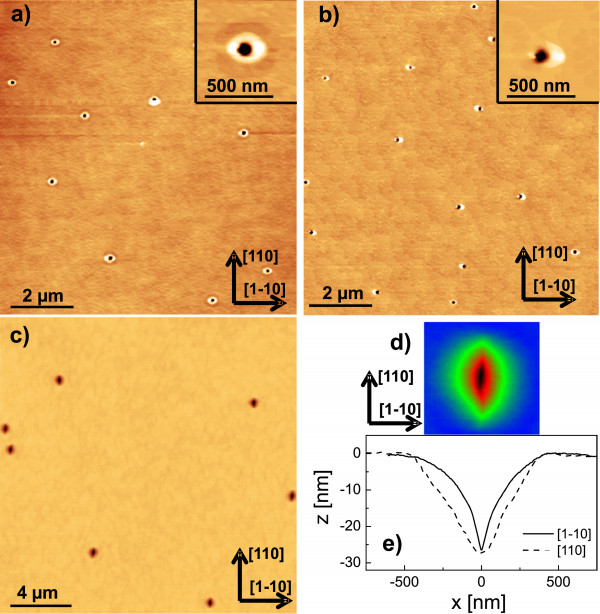
**AlGaAs surfaces after droplet etching, annealing, and overgrowth. (a)** AFM micrograph of an AlGaAs surface (35% Al content) after Ga droplet etching and 120-s annealing at *T* = 680℃. **(b)** AFM micrograph of an AlGaAs surface after Ga droplet etching and 1,800-s annealing at *T* = 680℃. **(c)** AFM micrograph of sample where large holes (see Figure 4) are overgrown with 20-nm AlGaAs (35% Al content). **(d)** Color-coded micrograph of a single hole from **(c)**. **(e)** AFM linescans of the hole from **(d)**.

The combined droplet/thermal etching process can, in principle, be integrated with heteroepitaxy. To explore this further, we consider an extension of the method to incorporate AlGaAs layers. As a first approach, hole formation in an AlGaAs layer with 35% Al content is investigated. For this, 2.0 ML Ga droplet material is deposited at *T* = 650℃ followed by annealing at the same temperature. Figure 7a shows an AFM micrograph of a reference sample with droplet etched holes but without long-time annealing (*t*_a_= 120 s). As a first point, we notice that the structural properties of the droplet etched holes depend on the substrate material. Nanoholes droplet etched on GaAs have a density of about *N* = 2 ×10^6^ cm ^−2^ and a depth of *d* = 68 nm (Figure 2d), whereas etching on AlGaAs under otherwise identical conditions yields *N* = 1.2 ×10^7^ cm ^−2^ and *d* = 20 nm. An AlGaAs sample with droplet etching and long-time annealing (*t*_a_= 1,800 s) is shown in Figure 7b. Obviously, no widening of the holes in AlGaAs is visible. The hole depth of *d* = 21 nm is unchanged by the long-time annealing within the measurement error, and only the shape of the wall around the hole opening has changed. We attribute this result to a higher thermal stability of AlGaAs in comparison to GaAs [28].

In a second approach, we have overgrown large widened holes with 20-nm AlGaAs (35% Al content). The large holes are prepared at *T* = 650℃ and *t*_a_= 1,800 s (see Figure 4a). After overgrowth, large holes are still visible (Figure 7c,d). AFM profiles (Figure 7e) show that the hole depth is reduced from 35 to 25 nm and that the overgrown holes are strongly elongated along the [110] direction. We have already demonstrated the fabrication of GaAs quantum dots with controlled size and shape by partial filling of symmetric LDE holes in AlGaAs [14,15]. Filling of holes shown in Figure 7c,d would suggest the possibility of creating elongated quantum dots, where polarized emission is expected.

## Conclusions

Long-time thermal annealing of nanoholes, formed initially in GaAs surfaces by Ga local droplet etching, leads to a substantial but controlled shape modification. The inverted cone-like droplet etched nanoholes are transformed during long-time annealing into significantly widened holes with flat bottoms and reduced depth. Therefore, the combined droplet/thermal etching process represents a fundamental extension of conventional droplet etching [1,6,13]. This is demonstrated, e.g. by strongly increased hole diameters of more than 1 *μ*m using droplet/thermal etching in comparison to conventional droplet etching with diameters of 50 to 200 nm [23]. The hole shape evolution can be qualitatively understood by a simplified model of mass transport by surface diffusion and direct evaporation. The hole diameter can be controlled by varying the annealing time or annealing temperature, offering a new means of manipulating hole morphology for possible applications as templates for nanostructure nucleation. Finally, in an initial approach, the integration of the combined droplet/thermal etching process with heteroepitaxy has been demonstrated.

## Competing interests

The authors declare that they have no competing interests.

## Authors’ contributions

CH conceived the study, fabricated some of the samples, performed AFM measurements and analysis, and prepared the manuscript draft. SS fabricated some of the samples and performed AFM measurements. DEJ developed a model to describe the experimental results and helped to draft the manuscript. WH participated in the study coordination and discussion of the results. All authors read and approved the final manuscript.
